# Down Syndrome Fetal Fibroblasts Display Alterations of Endosomal Trafficking Possibly due to SYNJ1 Overexpression

**DOI:** 10.3389/fgene.2022.867989

**Published:** 2022-05-13

**Authors:** Laura De Rosa, Dominga Fasano, Lucrezia Zerillo, Valeria Valente, Antonella Izzo, Nunzia Mollo, Giuseppina Amodio, Elena Polishchuk, Roman Polishchuk, Mariarosa Anna Beatrice Melone, Chiara Criscuolo, Anna Conti, Lucio Nitsch, Paolo Remondelli, Giovanna Maria Pierantoni, Simona Paladino

**Affiliations:** ^1^ Department of Molecular Medicine and Medical Biotechnology, University of Naples Federico II, Naples, Italy; ^2^ Department of Advanced Medical and Surgical Sciences, 2nd Division of Neurology, Center for Rare Diseases, University of Campania Luigi Vanvitelli, Naples, Italy; ^3^ Department of Medicine, Surgery and Dentistry “Scuola Medica Salernitana, University of Salerno, Salerno, Italy; ^4^ Telethon Institute of Genetics and Medicine, Pozzuoli, Italy; ^5^ Department of Neuroscience, Reproductive, and Odontostomatological Sciences, University of Naples Federico II, Naples, Italy; ^6^ Institute of Experimental Endocrinology and Oncology “G. Salvatore,” National Research Council, Naples, Italy

**Keywords:** Down syndrome, trisomy 21, endosomal trafficking, endolysosomal system, synaptojanin 1, membrane trafficking

## Abstract

Endosomal trafficking is essential for cellular homeostasis. At the crossroads of distinct intracellular pathways, the endolysosomal system is crucial to maintain critical functions and adapt to the environment. Alterations of endosomal compartments were observed in cells from adult individuals with Down syndrome (DS), suggesting that the dysfunction of the endosomal pathway may contribute to the pathogenesis of DS. However, the nature and the degree of impairment, as well as the timing of onset, remain elusive. Here, by applying imaging and biochemical approaches, we demonstrate that the structure and dynamics of early endosomes are altered in DS cells. Furthermore, we found that recycling trafficking is markedly compromised in these cells. Remarkably, our results in 18–20 week-old human fetal fibroblasts indicate that alterations in the endolysosomal pathway are already present early in development. In addition, we show that overexpression of the polyphosphoinositide phosphatase synaptojanin 1 (Synj1) recapitulates the alterations observed in DS cells, suggesting a role for this lipid phosphatase in the pathogenesis of DS, likely already early in disease development. Overall, these data strengthen the link between the endolysosomal pathway and DS, highlighting a dangerous liaison among Synj1, endosomal trafficking and DS.

## 1 Introduction

Down syndrome (DS) is a complex disorder with multiorgan involvement resulting from trisomy of chromosome 21 (Antonarakis et al., 2020). An increased dosage of chromosome 21 (Hsa21) genes is thought to cause the neuropathological and clinical manifestations of DS. The presence of more than 200 genes on Hsa21 implies that multiple cellular pathways may be altered and may therefore contribute to the pathogenesis of DS.

Endosomal trafficking is essential for cellular homeostasis. At the crossroads of exocytic and endocytic fluxes, the endolysosomal system is a critical hub for sorting molecules throughout the cell and for controlling the turnover of plasma membrane proteins (such as receptors, transporters, etc.), thus enabling to meet the demands of metabolism, growth, and environment (Maxfield and McGraw, 2004; Perret et al., 2005; Scott et al., 2014). The dysfunction of molecular machinery regulating endosomal trafficking leads to various diseases in humans (Schreij et al., 2016; Yarwood et al., 2020).

In the last decade, phosphoinositides, phosphorylated derivatives of phosphatidylinositol (PI), have emerged as fundamental regulators of membrane trafficking (Di Paolo and De Camilli, 2006; Vicinanza et al., 2008; Mayinger, 2012; Billcliff and Lowe, 2014). The proper concentration of these lipids is critical for the cells and is guaranteed by the activity of specific kinases and phosphatases, which in turn have to be finely controlled in space and time (Krauß and Haucke, 2007; Vicinanza et al., 2008; Balla, 2013). Among Hsa21 genes, SYNJ1, which maps very close to the putative DS critical region (Delabar et al., 1993; Korenberg et al., 1994; Shapiro, 1999) at 21q22.11 (UCSC Assembly GRCh38/hg38), encodes the ubiquitous inositol-phosphatase synaptojanin 1 (Synj1). Excessive expression of Synj1 in the postmortem brains of DS individuals was observed (Arai et al., 2002), supporting its involvement in DS.

With respect to other members of this class of enzymes, Synj1 harbors two consecutive phosphatase domains, Sac1 and 5-phosphatase, which dephosphorylates various substrates (McPherson et al., 1996; Guo et al., 1999). In particular, the Sac1 domain acts on the phosphatidylinositol monophosphates PI3P and PI4P that are enriched, respectively, at endosomes and Golgi membranes, while the 5-phosphatase domain dephosphorylates the PI4,5P2 on the plasma membrane.

Thanks to this double enzymatic activity, Synj1 is implicated in a wide range of cellular functions, even in dependence on the cell context. Firstly identified as the Grb2 interactor through overlay assays of synaptic fractions from rat brains (McPherson et al., 1994), the role of Synj1 in vesicle synaptic retrieval is widely established (McPherson et al., 1996; Haffner et al., 1997; Cremona et al., 1999; Harris et al., 2000; Mani et al., 2007). In addition, it is involved in the endocytosis of AMPA receptors postsynaptically (Gong and De Camilli, 2008), and its role in clathrin coat dynamics at synapses has also been reported (Cao et al., 2017).

Recently, a crucial role of Synj1 in regulating membrane trafficking has been shown in human cells (Fasano et al., 2018; Amodio et al., 2019; Choudhry et al., 2021). Remarkably, it has been demonstrated that Synj1 plays a key role in regulating the homeostasis and functions of early endosomes (EEs) in different human cell types, including neuronal cells (Fasano et al., 2018). In particular, loss of Synj1 activity leads to the enlargement of EEs and impairment of recycling trafficking (Fasano et al., 2018).

Interestingly, alterations of endosomal compartments were observed in mononuclear blood and lymphoblastoid cells and in fibroblasts from adult individuals with DS (Cossec et al., 2012; Botté et al., 2020), suggesting that the dysfunction of the endosomal pathway may concur to the pathogenesis of DS.

Here, we applied quantitative confocal microscopy and biochemical assays to analyze the endosomal trafficking and the homeostasis of the endolysosomal system. Remarkably, our findings in 18–20 week-old human fetal fibroblasts indicate that alterations in the endolysosomal pathway are already present early in development.

## 2 Materials and Methods

### 2.1 Ethics Statement

Cultures of human fetal fibroblasts from fetuses with trisomy of Hsa21 and from euploid fetuses at 18–20 gestational weeks were obtained from the “Telethon Bank of Fetal Biological Samples” at the University of Naples. All experimental protocols were approved by the local Institutional Ethics Committee. Specifically, five euploid and seven trisomic cultures were used.

### 2.2 Reagents and Antibodies

Primary antibodies included the following: rabbit polyclonal anti-Cathepsin D (Thermo Fisher Scientific), rabbit polyclonal anti-EEA1 (Thermo Fisher Scientific), rabbit polyclonal anti-FGF receptor 1 (Cell Signaling Technology), mouse monoclonal anti-Lamp-1 (BD Pharmingen™), mouse monoclonal anti-Rab5 (BD Transduction Laboratories™), mouse monoclonal anti-Rab7 (Santa Cruz Biotechnology, Inc.), and rabbit polyclonal anti-Synj1 (Abcam plc). LysoTracker^®^ Red DND-99 and fluorescent transferrin conjugates were from Molecular Probes (Thermo Fisher Scientific); human basic fibroblast growth factor (hFGF basic/FGF2) was from Cell Signaling Technology. Alexa Fluor secondary antibodies were from Thermo Fisher Scientific, and horseradish peroxidase (HRP)-conjugated secondary antibodies used for Western blot analysis were from GE Healthcare.

### 2.3 Cell Cultures and Transfections

Fibroblasts were grown in Dulbecco’s modified Eagle’s medium (DMEM) (Merck Life Science) supplemented with 2 mM l-glutamine, 20% FBS (Thermo Fisher Scientific), and 1% penicillin/streptomycin (Thermo Fisher Scientific). All experiments described throughout this study were carried out on five euploid and seven trisomic primary cultures at passages 4–5.

SH-SY5Y cells were maintained in RPMI-1640 with 10% fetal bovine serum (FBS) and 2 mM l-glutamine. All cells were maintained at 37°C in a saturated humidity atmosphere containing 95% air and 5% CO_2_.

Synj1 overexpression was obtained by transfecting SH-SY5Y cells with a plasmid vector, pCMV6-SYNJ1, encoding human SYNJ1 (Myc-DDK-tagged; OriGene Technologies). Transfection was performed using the XtremeGene-9 DNA Transfection Reagent (Merck Life Science) according to the manufacturer’s protocol. Stably transfected cells were obtained after selection with geneticin (0.5 μg/ml, Thermo Fisher Scientific); in particular, we collected both pools of clones or single clones derived from single cell colonies.

SYNJ was transiently silenced in two DS-HFF lines using specific siRNA (ID: s16934) and a non-targeting negative control siRNA with the same chemical modifications was used as negative control (from Ambion by Life Technologies). Interferin transfection reagent (Polyplus) was used. Cells were plated on 6-well plates (300,000 cells/well), also containing coverslips for immunofluorescence assays, and transfected with 20 nM siRNA according to the manufacturer’s protocol. Seventy-2 hours after transfection, the effects of SYNJ1 siRNA-mediated attenuation were evaluated.

### 2.4 RNA Extraction and Quantitative RT-PCR

Total RNA isolation was performed using the TRI-reagent solution (Merck Life Science). cDNA was synthesized from 1 μg of total RNA using the QuantiTect Rev. Transcription Kit (Qiagen). qRT-PCR was performed using the FluoCycle Master Mix (Euroclone). All these procedures were conducted according to manufacturer’s instructions as previously described (Pierantoni et al., 2003; Conte et al., 2017). Gene-specific primers used for amplification were as follows:

*Synj1*-Fw: 5′-CAA​CCC​GAT​ACC​ATC​GGA​CA-3′;
*Synj1*-Re: 5′- TGT​TTC​CAG​AGA​TCT​CCC​CG-3′;
*Actin*-Fw: 5′- CTA​AGG​CCA​ACC​GTG​AAA​AG -3′;
*Actin*-Re: 5′- ACCAGAGGCA TACAGGGACA -3′.

The relative expression levels were calculated by using the 2^−ΔΔCT^ formula.

### 2.5 Western Blot Analysis

Fibroblasts were lysed with JS lysis buffer (Hepes pH 7.5 50 mM, NaCl 150 mM, glycerol 1%, Triton X-100 1%, MgCl_2_ 1.5 mM, and EGTA 5 mM) containing a cocktail of protease inhibitors (Merck Life Science). Lysates were run on sodium dodecyl sulfate–polyacrylamide gel electrophoresis, transferred onto polyvinylidene difluoride or nitrocellulose membranes, revealed by Western blotting using specific antibodies and then detected with HRP-conjugated antibodies.

### 2.6 Fluorescence Microscopy

Cells, grown on coverslips, were washed with phosphate-buffered saline (PBS), fixed with 4% paraformaldehyde (PFA), and quenched with 50 mM NH_4_Cl. Then, cells were permeabilized with 0.2% Triton X-100 for 5 min and blocked for 30 min in PBS containing 2% FBS and 0.2% bovine serum albumin (BSA). For Lamp-1 immunolabeling, cells were fixed with 100% ice-cold methanol for 5 min for preserving lysosomal morphology as recently shown (Scerra et al., 2021); then blocked for 30 min in PBS containing 2% FBS and 0.2% BSA. Primary antibodies were detected with Alexa Fluor-conjugated secondary antibodies.

For lysosome staining, cells were incubated for 1 h at 37°C with 1 μM LysoTracker in complete medium before fixing.

Images were collected using the LSM 700 confocal laser scanning microscope (Carl Zeiss, Jena, Germany) equipped with a Plan Apo ×63 oil immersion objective lens (NA 1.4); diode lasers (at 405, 488, and 555 nm) were used as light sources. Images were acquired with the confocal pinhole set to one Airy unit using the same setting (laser power, detector gain, and threshold of fluorescence intensity) in all experimental conditions. Three-dimensional reconstructions of Z-slices collected from the top to the bottom of the cells were carried out using Zeiss ZEN Black software, as well as quantification analyses. In particular, the mean fluorescence intensities were measured by drawing regions of interest (ROIs) around the entire cell as previously described (Fasano et al., 2018).

### 2.7 Internalization Assays

#### 2.7.1 Tf Internalization Assay

To monitor Tf internalization and recycling, cells were incubated with Alexa Fluor-546-conjugated Tf (10 μg/ml) in culture medium containing 0.5% BSA for 7 min at 37°C (pulse), washed to remove the excess of unbound Tf and chased for different times (10 and 20 min). Then, cells were fixed with 4% PFA.

#### 2.7.2 FGFR Internalization Assay

The cells were serum starved for 16 h to prevent ligand-dependent internalization (Irschick et al., 2013). In order to exclusively analyze the trafficking of proteins coming from the surface, cells were incubated with cycloheximide (150 μg/ml) for at least 3 h to avoid new protein synthesis. Then, cells were stimulated with FGF (10 ng/ml) at 37°C, lysed at different times and subjected to Western blot analysis. The same experimental procedure was followed for immunofluorescence experiments. Cells were fixed with ice-cold methanol.

### 2.8 Electron Microscopy

Cells were fixed with a mixture of 4% PFA and 0.05% glutaraldehyde (GA) for 10 min at room temperature, then washed with 4% PFA once to remove the residual GA and fixed again with 4% PFA for 30 min at room temperature. Next, cells were incubated with a blocking/permeabilizing mixture (0.5% BSA, 0.1% saponin, 50 mM NH_4_Cl) for 30 min and subsequently with the primary monoclonal antibody against Lamp-1, diluted 1:500 in blocking/permeabilizing solution. The following day, cells were washed and incubated with the secondary antibody, the anti-mouse Fab fragment coupled to 1.4 nm gold particles (diluted 1:50 in blocking/permeabilizing solution) for 2 h at room temperature. Cells were then post-fixed as described in Polishchuk et al., 2019. After dehydration, specimens were embedded in epoxy resin and polymerized at 60°C for 72 h. Thin 60 nm sections were cut on a Leica EM UC7 microtome. EM images were acquired from thin sections using a FEI Tecnai-12 electron microscope equipped with a VELETTA CCD digital camera (FEI, Eindhoven, the Netherlands). Morphometric analysis on the size of lysosome-like structures was performed using iTEM software (Olympus SYS, Germany).

### 2.9 Statistical Analysis

The two-tailed Student’s *t*-test was used for statistical analysis.

## 3 Results

### 3.1 The Homeostasis of Early Endosomes Is Impaired in DS Fetal Fibroblasts

To test whether endosomal trafficking was altered in the early stages of DS, we analyzed human fibroblasts derived from fetuses with DS of 18–20 gestational weeks (DS-HFFs) in comparison with their non-trisomic counterparts (EU-HFFs).

The endolysosomal pathway relies on pleomorphic and highly dynamic membrane compartments with a specific molecular identity, regarding both membrane and luminal components. Combining imaging and biochemical approaches, we analyzed the morphology and dynamics of the endolysosomal compartments.

**FIGURE 1:**
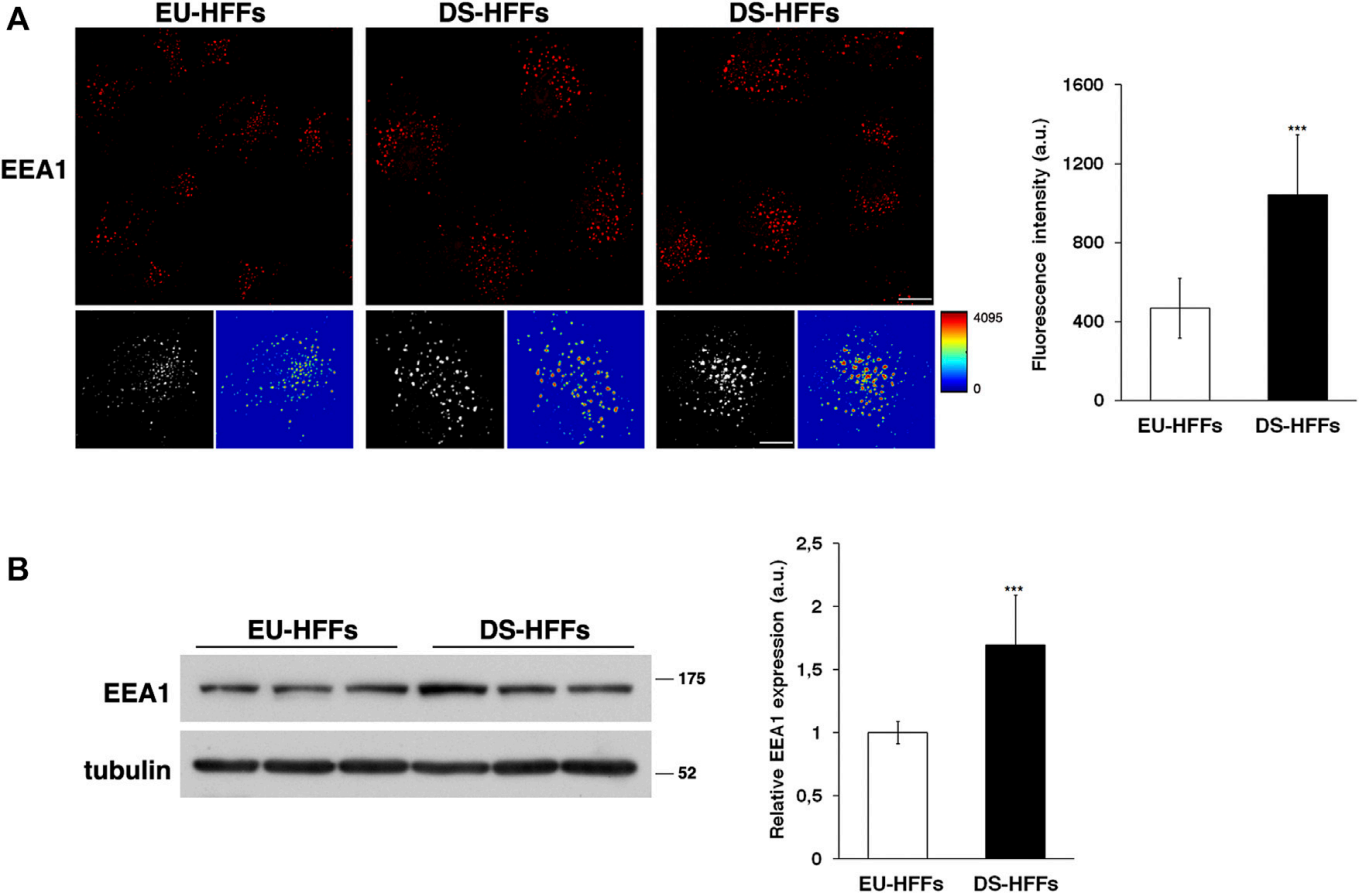
The homeostasis of early endosomes is altered in DS fetal fibroblasts. **(A)** Human fetal fibroblasts from euploid (EU-HFFs) and DS (DS-HFFs) fetuses were stained with early endosome antigen 1 (EEA1) antibody detected with Alexa-546-conjugated secondary antibodies. Serial confocal sections were collected from the top to the bottom of the cells. Representative images showing that the early endosomes are enlarged in trisomic cells when compared to euploid cells. For each condition, the 3D reconstruction and corresponding intensity map are shown. Scale bars, 8 μm. The mean fluorescence intensity (arbitrary unit, a.u.) of EEA1-positive structures is higher in DS-HFFs than in EU-HFFs. The bars show the relative mean value ± SD of three independent experiments, *n* ≥ 50 cells. **(B)** Representative immunoblotting of EEA1 in DS-HFFs and EU-HFFs. Tubulin was used as the loading control. The molecular weight of protein markers is indicated. Densitometric analysis of three different experiments, performed in three euploid and in four trisomic cultures, is shown. Error bars, mean ± SD. ***p < 0.001, Student’s *t*-test.

First, early endosomes (EEs) were labeled by immunofluorescence with an antibody against a specific marker of these organelles, early endosome antigen 1 (EEA1). By confocal microscopy analysis, we observed remarkable changes in the structure of EEs in DS-HFFs compared with EEs in EU-HFFs (Figure 1A). The EEA1-positive structures of DS-HFFs were larger, as clearly evident in 3D reconstructions, and displayed higher fluorescence intensity with respect to euploid cells (Figure 1A), indicating an enlargement of these compartments. Interestingly, higher levels of EEA1 were observed in trisomic cells by Western blot analysis (Figure 1B), suggesting that EEA1 is stalled on the compartment.

**FIGURE 2:**
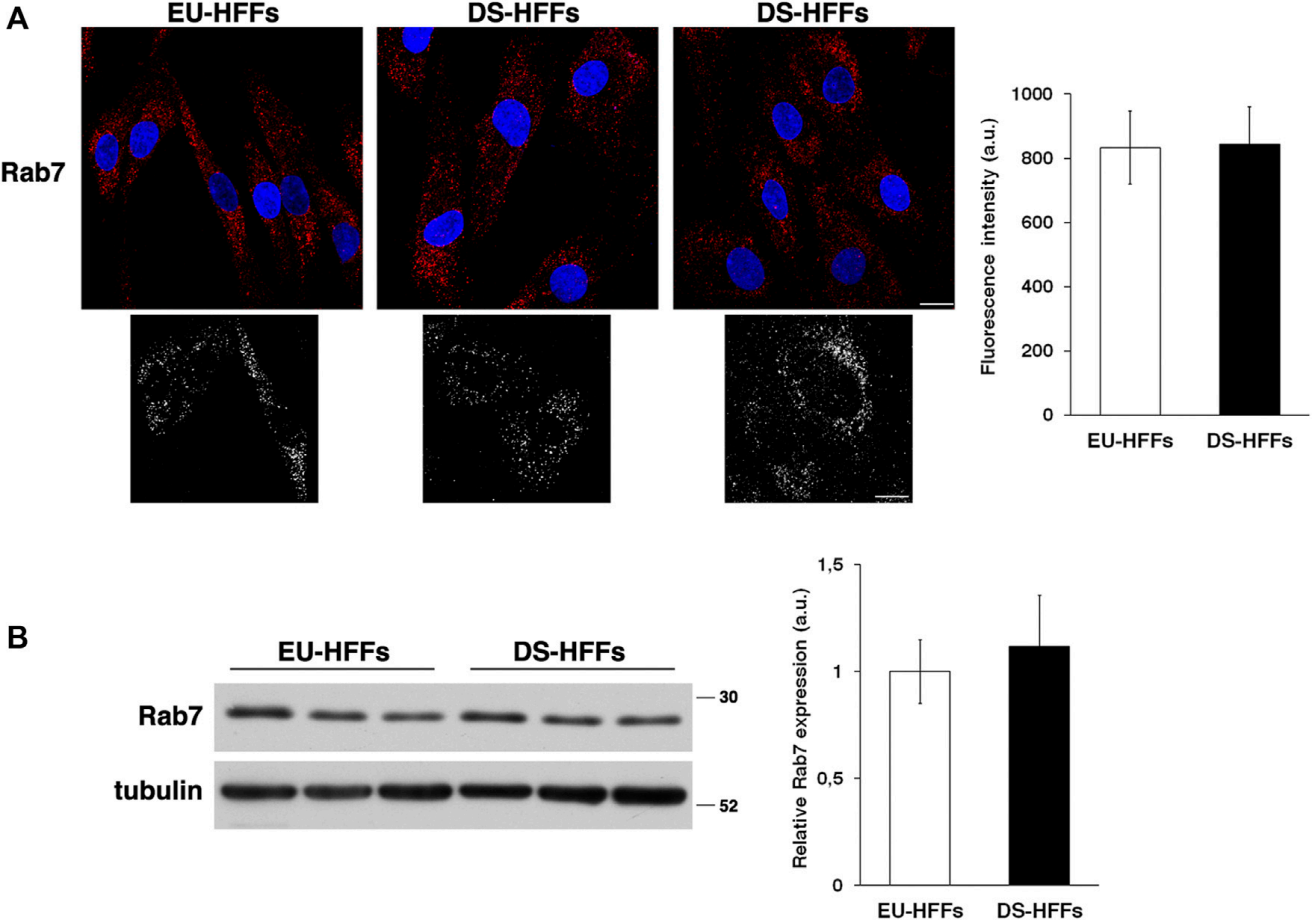
The homeostasis of late endosomes is not altered in DS-HFFs. **(A)** EU-HFFs and DS-HFFs were stained with Rab7 antibody detected with Alexa-546-conjugated secondary antibodies. Serial confocal sections were collected from the top to the bottom of the cells. Representative images showing comparable late endosomes in both euploid and trisomic cells. For each condition, the 3D reconstruction is shown. Scale bars, 8 μm. Mean fluorescence intensity (arbitrary unit, a.u.) of Rab7-positive structures is shown. The bars show the relative mean value ± SD of three independent experiments, *n* ≥ 50 cells. **(B)** Representative immunoblotting of Rab7 in DS-HFFs and EU-HFFs. Tubulin was used as the loading control. The molecular weight of protein markers is indicated. Densitometric analysis of three different experiments, performed in three euploid and trisomic cultures, is shown. Error bars, mean ± SD.

In contrast, no major alterations of the late endosomes stained with an anti-Rab7 antibody were observed (Figure 2A). Consistently, we found comparable levels of Rab7 in EU-HFFs and DS-HFFs by immunoblot experiments (Figure 2B).

Altogether, these data indicate that the homeostasis of EEs is strongly impaired in trisomic cells.

Together with previous findings showing an enlargement of early endosomal compartments in cells from adults with DS (Cossec et al., 2012; Botté et al., 2020), our data further support the involvement of the endosomal pathway in DS. Moreover, they also highlight that endosomal alterations possibly occur early during development.

### 3.2 The Dynamics of Early Endosomes Is Impaired in DS Fetal Fibroblasts

The small GTPase Rab5 is the master regulator of EE biogenesis and dynamics (Bucci et al., 1992; Rybin et al., 1996; Stenmark, 2009; Zeigerer et al., 2012; Murray et al., 2016).

**FIGURE 3:**
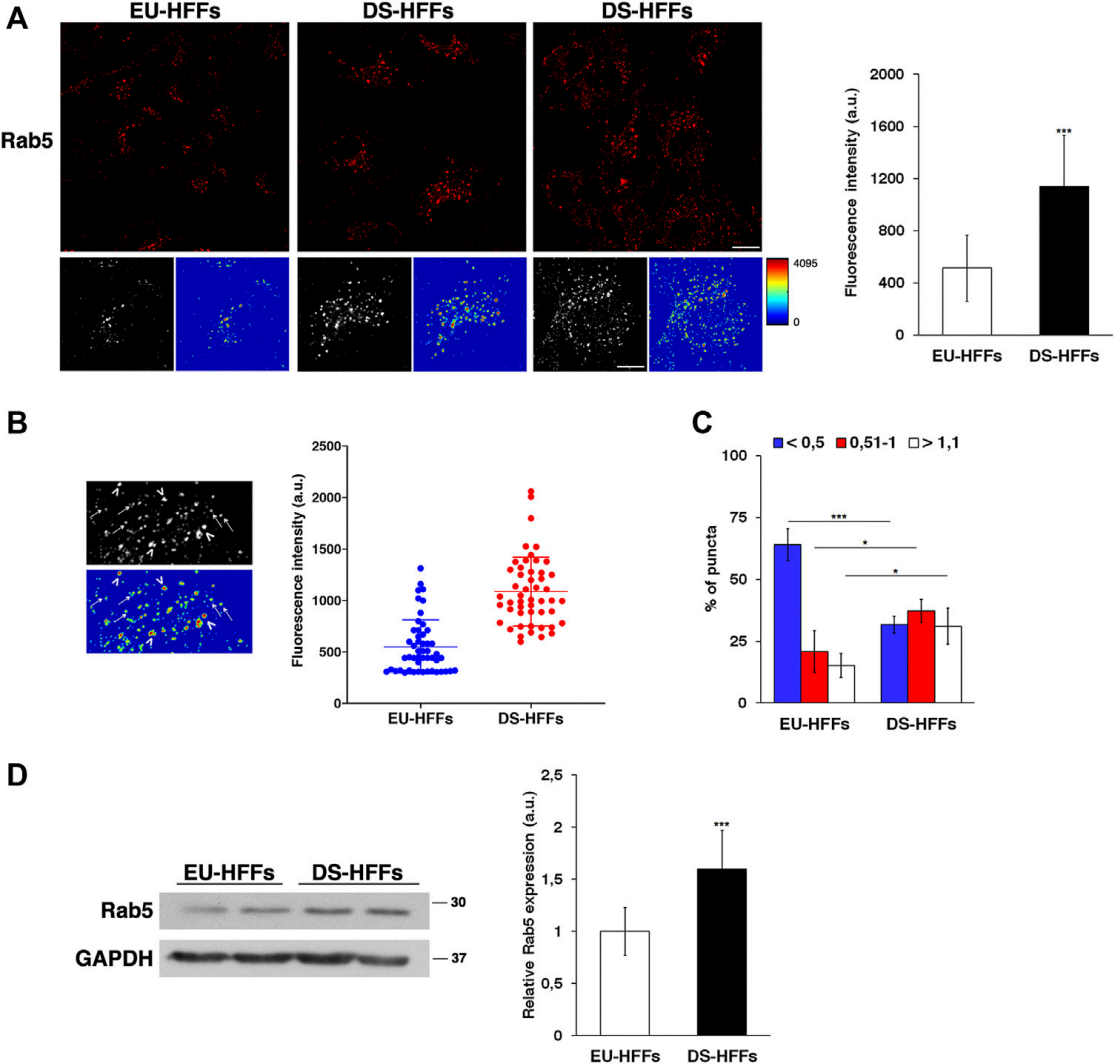
The dynamics of early endosomes is perturbed in DS-HFFs. **(A)** EU-HFFs and DS-HFFs were stained with Rab5 antibody detected with Alexa-546-conjugated secondary antibodies. Serial confocal sections were collected from the top to the bottom of the cells. For each condition, the 3D reconstruction and corresponding intensity map are shown. Scale bars, 8 μm. Mean fluorescence intensity (arbitrary unit, a.u.) of Rab5-positive structures is higher in DS-HFFs than in EU-HFFs. The bars show the relative mean value ± SD of three independent experiments. **(B)** Representative 3D reconstruction and corresponding intensity map of cell ROIs taken into analysis (left panels). Two populations of Rab5 endosomes are distinguishable in terms of brightness, with lower (arrows) and higher (arrowheads) intensity fluorescent signal. Representative scatter dot plot showing the distribution of fluorescence intensity of Rab5-positive structures in EU-HFFs and DS-HFFs (right panel). Each dot represents one sample value, error bars represent mean value ± SD. **(C)** The percentage of larger Rab5 positive structures (>1 μm) are increased in DS-HFFs when compared to that in EU-HFFs. The bars show the relative mean value ± SD of three independent experiments, *n* ≥ 30 cells. **(D)** Representative immunoblotting of Rab5 in DS-HFFs and EU-HFFs. GAPDH was used as the loading control. The molecular weight of protein markers is indicated. Densitometric analysis of three different experiments, performed in three euploid and in four trisomic cultures, is shown. Error bars, mean ± SD. *p < 0.05; ***p < 0.001, Student’s *t* -test.

Therefore, we performed imaging analysis of EEs by labeling them with an anti-Rab5 antibody. Similar to what we observed with EEA1 staining, the structure of Rab5-positive compartments was strikingly altered in DS-HFFs - they occupied a larger area of the cell as evident in 3D reconstructions and their mean fluorescence intensity was significant higher (Figure 3A).

In addition, it is worth noting that the brightness profile of Rab5-positive dots was heterogeneous as evidenced in the intensity maps (Figures 3A,B). In contrast from EU-HFFs, in DS-HFFs, the fluorescence-intensity distribution analysis showed the existence of two subpopulations, one with very high intensity fluorescent signals (Figure 3B), providing evidence for the altered dynamics of EEs in DS cells. Furthermore, the endosome size was significantly increased in DS-HFFs with respect to EU-HFFs (Figure 3C); indeed, the percentage of larger Rab5-positive structures was greater in DS-HFFs (Figure 3C).

In agreement with imaging data, Western blot analysis showed higher levels of Rab5 in trisomic cells compared with euploid cells (Figure 3D), further supporting its altered dynamics.

Overall, all these data clearly provide evidence that the homeostasis and dynamics of EEs are altered in DS.

### 3.3 Recycling Trafficking Is Perturbed in DS Fetal Fibroblasts, but Trafficking to Lysosomes Is Not

EEs are a central sorting station along the endocytic pathway, controlling the reutilization or the degradation of internalized molecules.

To test whether endosomal trafficking is affected in DS, we examined transferrin (Tf) receptor and fibroblast growth factor (FGF) receptor 1 (FGFR1) trafficking, which follow two distinct fates upon internalization: recycling to the plasma membrane and targeting to lysosomes for degradation.

**FIGURE 4:**
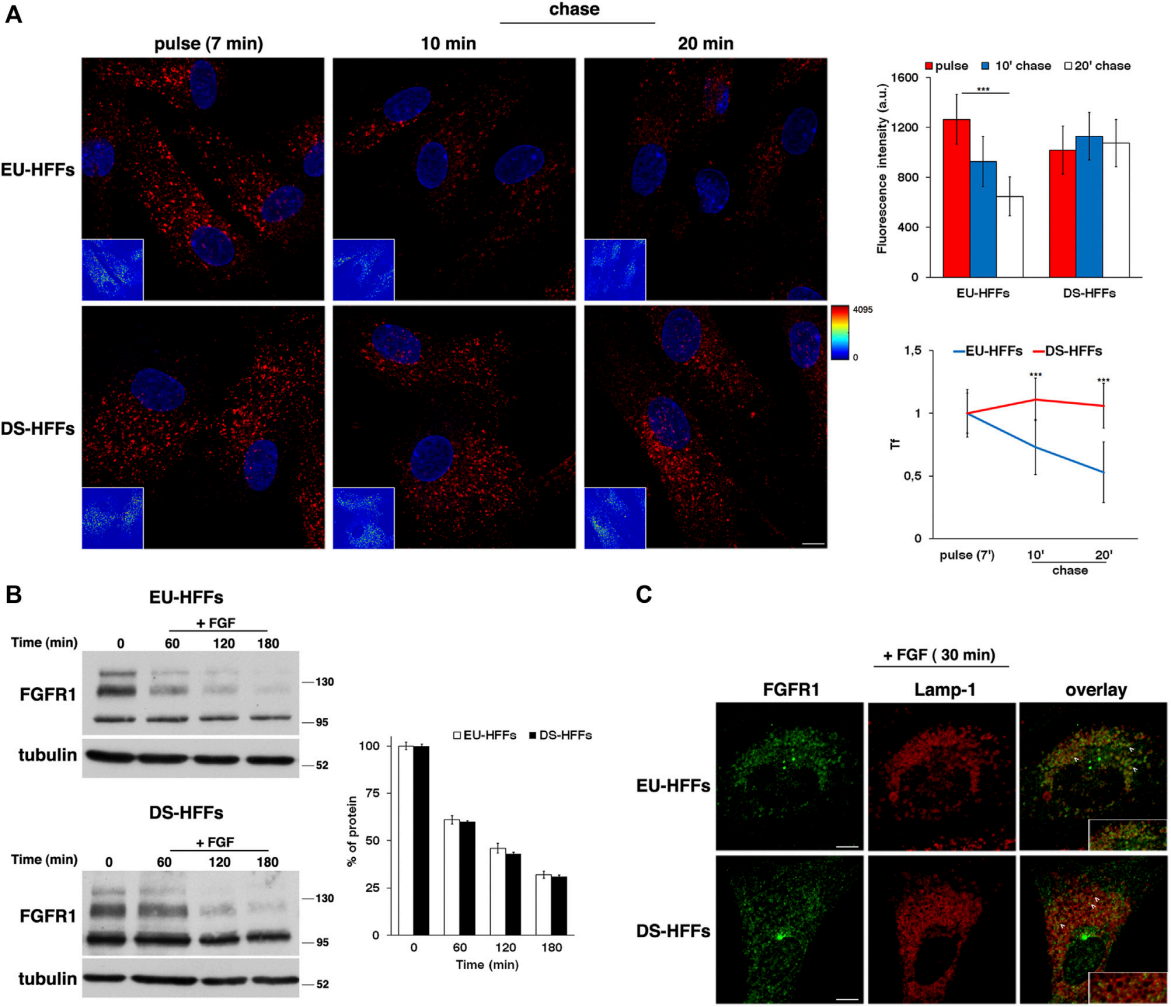
The recycling trafficking, but not the traffic toward lysosomes, is perturbed in DS-HFFs. **(A)** The internalization assay of Alexa-546-conjugated transferrin (Tf) in EU-HFFs and DS-HFFs. Tf was added to the cells at 37°C for 7 min (pulse). After washing out, cells were incubated in culture medium for different indicated times (chase). Representative confocal images and corresponding intensity maps in the insets (enlarged pictures in Supplementary Figure S1) are shown; scale bars, 8 μm. Note that the fluorescent signal of Tf decreased over time in EU-HFFs, while it remained constant in DS-HFFs. Mean fluorescence intensity (arbitrary unit, a.u.) of Tf is shown (upper graph); error bars, mean ± SD of three independent experiments, *n* ≥ 40 cells. Curves of Tf internalization expressed as mean values of fluorescence measured at chase times compared with the fluorescent signal after the pulse (set to 1) are shown (lower graph). **(B)** Representative immunoblotting of FGFR1 upon FGF stimulation. Cells were serum starved for 16 h (time) and incubated with FGF (10 ng/ml) for different times (see *Materials and Methods*). Tubulin was used as the loading control. The molecular weight of protein markers is indicated. The three bands detected for FGFR1 in both euploid and trisomic cells represent two glycosylated, mature and immature, forms (upper and intermediate band, respectively) and a non-glycosylated form (lower band), as previously described (Ying et al., 2020). Densitometric analysis of three independent experiments is shown, and the results are expressed as a percentage of the amount of protein at time 0. Error bars, mean ± SD. **(C)** Cells treated as in **(B)** were fixed and stained with FGFR1 (green) and Lamp-1 (red) antibodies. Representative images corresponding to 30 min of FGF incubation are shown; scale bars, 8 μm. After internalization, FGFR1 localized in cytoplasmic punctate structures, some of which were co-localized with Lamp-1 positive dots (see arrows). A region of cell at higher magnification is shown in the insets. ***p < 0.001, Student’s *t*-test.

We monitored the trafficking of fluorescent Alexa-546 conjugated Tf by performing pulse-chase based endocytic assays as previously described (Fasano et al., 2018). After incubation with fluorescent Tf for 7 min at 37°C (pulse), cells were washed to remove the unbound Tf and incubated in culture medium for different times at 37°C (chase). A progressive decrease of Tf signal was observed at the chase times in EU-HFFs (Figure 4A). In contrast, in DS-HFFs, the fluorescent signal remained higher at the same chase timepoints as clearly evidenced by quantitative analysis (Figure 4A), indicating a delay and/or impairment of the recycling pathway.

To study the dynamics of endocytosed FGFR1, cells were serum starved for 16 h in order to prevent ligand-dependent internalization and then incubated with FGF (10 ng/ml) at 37°C for different time points. We used cycloheximide, an inhibitor of protein synthesis, in the last 3 h of starvation and during FGF induction to exclusively follow the internalization of the FGFR1 pool accumulated at the plasma membrane. We evaluated the levels of FGFR1 immediately after starvation (time 0) and after FGF addition (+FGF) by Western blot analysis (Figure 4B).

After FGF induction, the levels of FGFR1 decreased to the same extent and with comparable kinetics in EU-HFFs and DS-HFFs (Figure 4B). In agreement with the biochemical data, after 30 min of stimulation, FGFR1 displayed a comparable distribution in Lamp-1-positive compartments in both euploid and trisomic cells (Figure 4C, see arrowheads). Altogether, these results indicate that trafficking to lysosomes was not affected in DS cells.

### 3.4 The Excessive Expression of Synj1 Affects the Homeostasis of EEs and Perturbs the Trafficking of Transferrin Receptor

The findings described above prompted us to test whether the endosomal abnormalities observed in DS cells were the result of Synj1 overexpression, considering its role in controlling the homeostasis and functions of EEs (Fasano et al., 2018).

**FIGURE 5:**
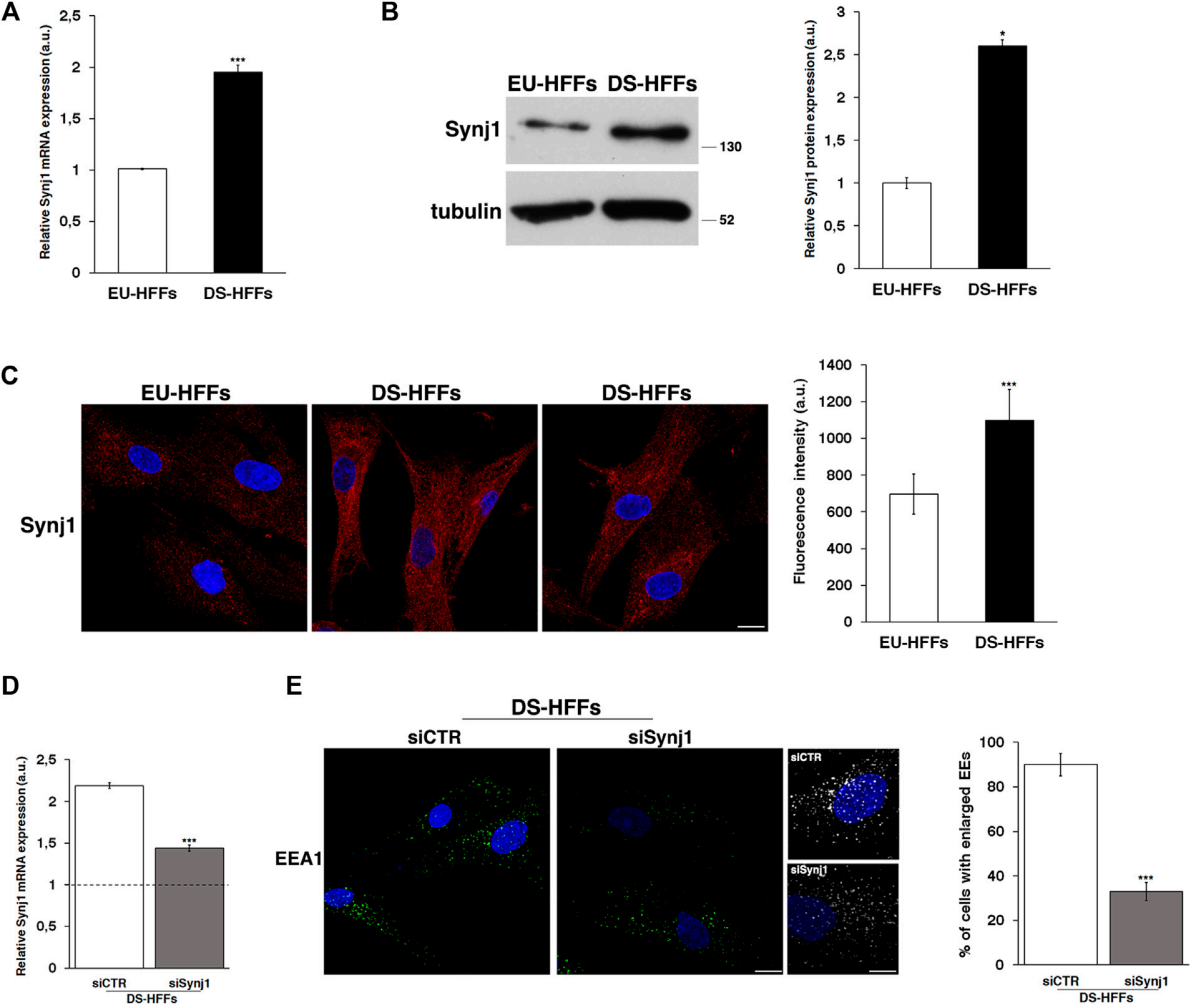
Synj1 is overexpressed in fetal trisomic fibroblasts. **(A)** Synj1 mRNA expression in EU-HFFs and DS-HFFs was analyzed by qRT-PCR. Results are presented as the mean ± SD of three independent experiments performed in triplicate. **(B)** Representative immunoblotting of Synj1 in EU-HFFs and DS-HFFs. Tubulin was used as the loading control. The molecular weight of protein markers is indicated. Densitometric analysis of three different experiments is shown. Error bars, means ± SD. **(C)** Immunostaining of Synj1 in EU-HFFs and DS-HFFs is shown. Scale bar, 8 μm. Mean fluorescence intensity (arbitrary unit, a.u.) of Synj1 is higher in DS-HFFs than in EU-HFFs. **(D)** DS-HFFs were transiently transfected with scrambled (siCTR) or specific anti-Synj1 (siSynj1) siRNA and relative Synj1 mRNA expression was measured by qRT-PCR. Results are presented as the mean ± SD of two independent experiments performed in triplicate. **(E)** Early endosomes of siCTR and siSynj1 DS-HFFs were labelled with EEA1 antibody, detected with Alexa-488-conjugated secondary antibodies. Serial confocal sections were collected from the top to the bottom of the cells. Representative images and corresponding 3D reconstructions showing that the attenuation of Synj1 restores the EEs aberrant phenotype. Scale bars, 8 μm. The graph shows the percentage of cells displaying enlarged EEA1-positive dots. The bars show the relative mean value ± SD of two independent experiments, *n* ≥ 30 cells. *p < 0.05; ***p < 0.001, Student’s *t*-test.

First, we analyzed the mRNA and protein expression of Synj1 in trisomic and euploid cells (Figures 5A,B), showing that Synj1 mRNA levels were significantly increased (∼2-fold) in DS-HFFs compared with EU-HFFs (Figure 5A). Consistently, protein expression was significantly upregulated, as shown by Western blot (Figure 5B) and immunofluorescence (Figure 5C) assays. The distribution of Synj1 was comparable in euploid and trisomic cells, and it was localized in the cytoplasm, preferentially in discrete structures resembling endocytic compartments (Figure 5C).

We then analyzed whether reducing Synj1 expression in DS fibroblasts would decrease the proportion of enlarged EEs. To this aim, DS fibroblasts were transiently transfected with specific RNAi against Synj1 as previously described (Fasano et al., 2018), and EEA1 immunolabelling was performed 72 hours after transfection, that is when the levels of mRNA resulted reduced about 1.5 fold with respect to control interfered cells (Figure 5D). Attenuation of Synj1 expression was able to partially restore the EE morphology (Figure 5E) as shown by the reduced number of cells with enlarged EEs with respect to control interfered cells (Figure 5E).

**FIGURE 6:**
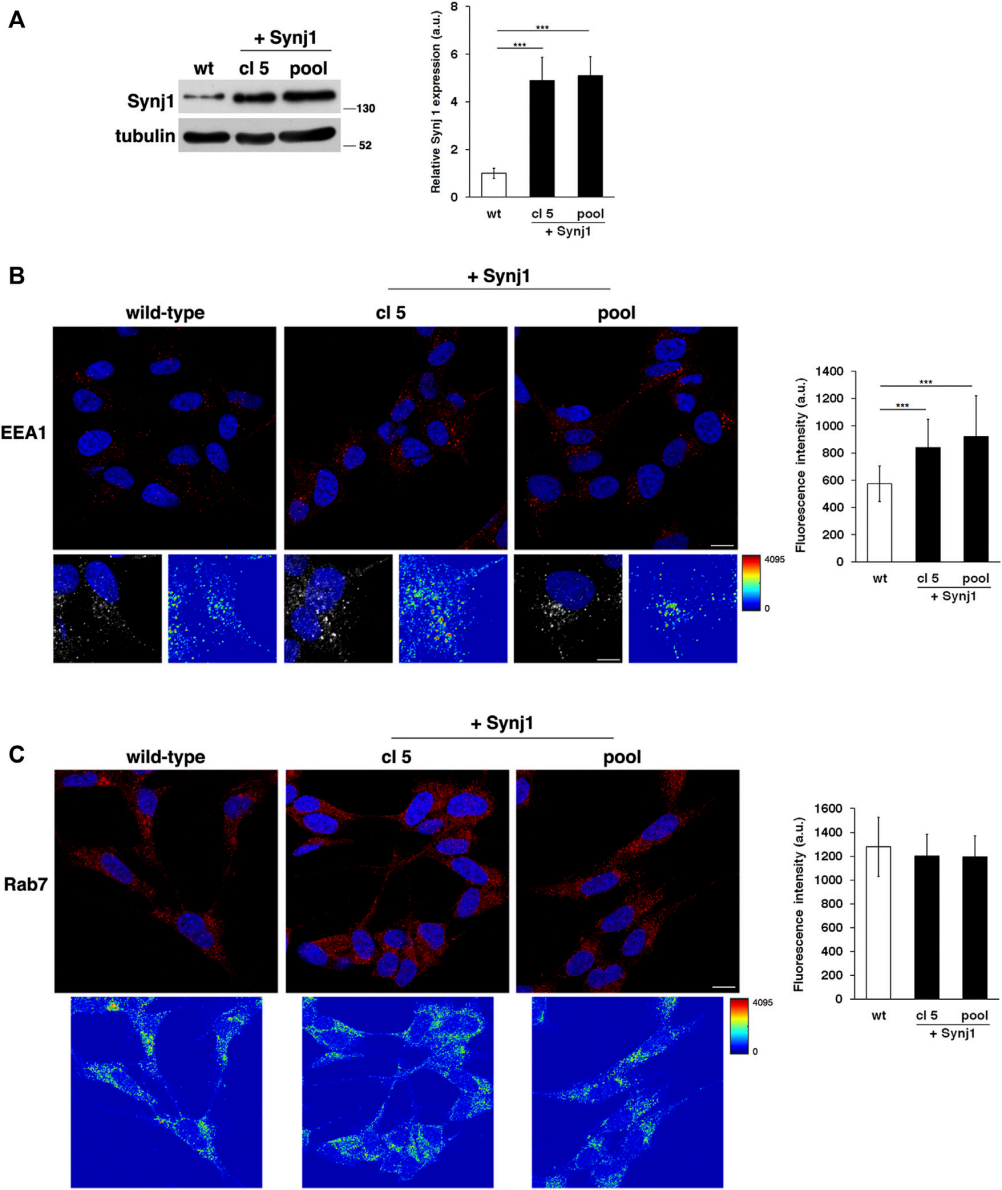
Synj1 overexpression affects the homeostasis of early endosomes, but not of late endosomes, in SH-SY5Y cells. **(A)** SH-SY5Y cells stably transfected with cDNA encoding for wild-type Synj1 were tested for the expression of Synj1 by Western blotting. Representative immunoblotting of Synj1 in a single clone (cl5) and in a pool of clones (pool) is shown. Tubulin was used as the loading control. The molecular weight of protein markers is indicated. Densitometric analysis of three different experiments is shown. Error bars, mean ± SD. **(B)** Cells were stained with EEA1 antibody detected with Alexa-546-conjugated secondary antibodies. Serial confocal sections were collected from the top to the bottom of the cells; scale bars, 8 μm. For each condition, the 3D reconstruction and corresponding intensity map are shown; scale bars, 5 μm. The mean fluorescence intensity (arbitrary unit, a.u.) of EEA1-positive structures is higher in Synj1-overexpressing than in wild-type cells. The bars show the relative mean value ± SD of three independent experiments, *n* ≥ 50 cells. **(C)** Immunostaining of Rab7 in wild-type and Synj1-overexpressing cells is shown. For each condition, the corresponding intensity map is shown. Scale bars, 8 μm. Mean fluorescence intensity (arbitrary unit, a.u.) is shown. The bars show the relative mean value ± SD of three independent experiments, *n* ≥ 50 cells. ***p < 0.001, Student’s *t* -test.

Next, to directly test the contribution of Synj1, we evaluated the effects of its overexpression on the endosomal pathway. To this aim, we stably transfected human neuroblastoma derived SH-SY5Y cells with a plasmid vector encoding Synj1 (see *Materials and Methods*). We obtained several pools of clones and single clones in which the expression of Synj1 was increased (Figure 6A).

**FIGURE 7:**
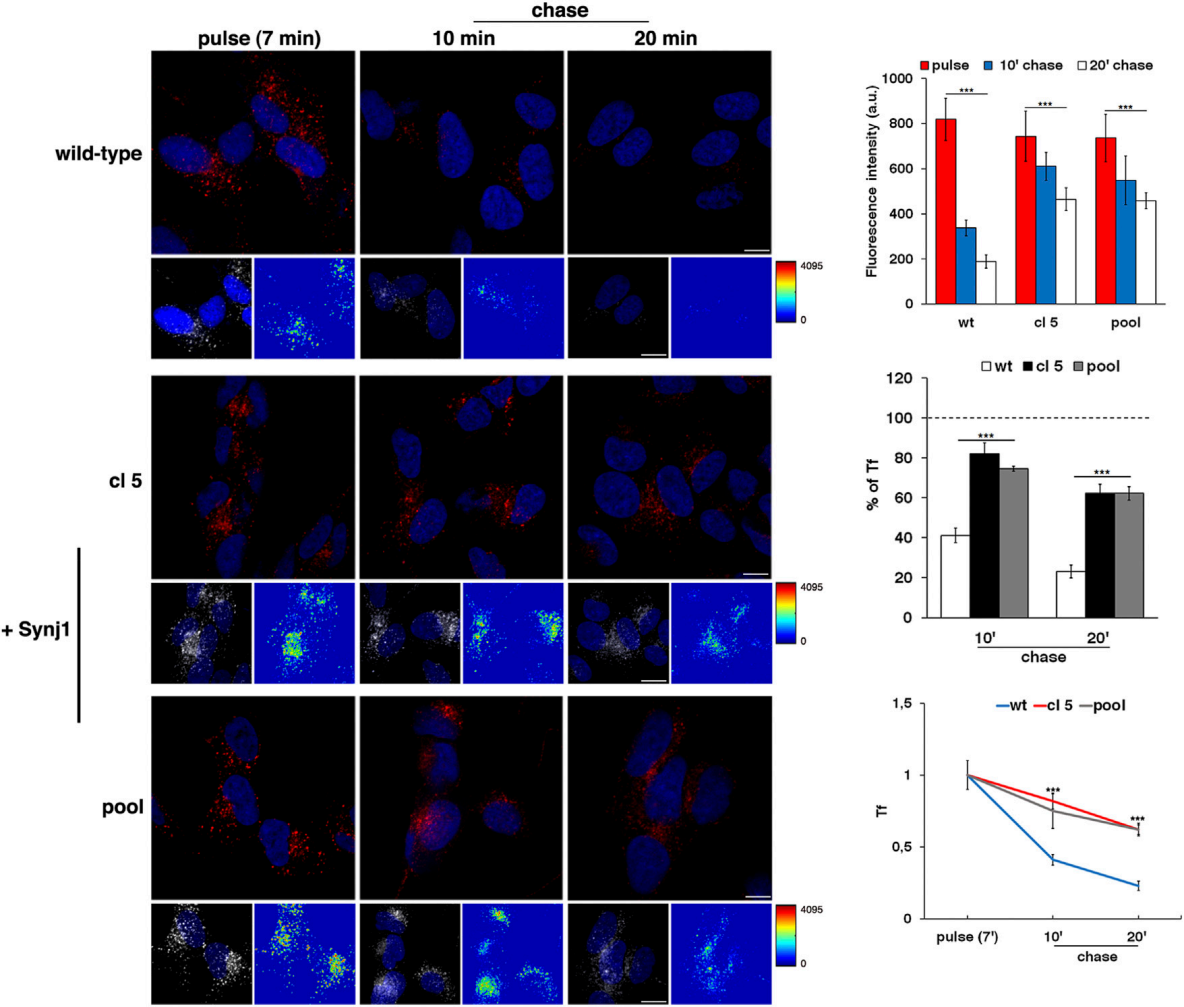
The recycling trafficking is perturbed in Synj1-overexpressing SH-SY5Y cells. The internalization assay of Alexa-546-conjugated transferrin (Tf) in wild-type and Synj1-overexpressing (+Synj1) SH-SY5Y cells was performed as in Figure 4A. Representative confocal images, 3D reconstruction of an area of the field and the corresponding intensity maps are shown; scale bars, 8 μm. Mean fluorescence intensity was measured at the different time points and expressed as arbitrary unit (a.u.; upper graph) or as the percentage of fluorescence detected at the pulse (set to 100%) (middle graph); error bars, mean ± SD of two independent experiments, *n* ≥ 50 cells. Curves of Tf internalization expressed as mean values of fluorescence measured at chase times compared with the fluorescent signal after the pulse (set to 1) are shown (lower graph). ***p < 0.001, Student’s *t-test*.

As revealed by the EEA1 antibody, Synj1 overexpression induced the expansion of the EEs. Indeed, the EEA1-positive compartments were larger and had higher fluorescent signals in Synj1-overexpressing SH-SY5Y cells compared with wild-type cells (Figure 6B). Comparable results were obtained for Rab5 (Supplementary Figure S2). On the contrary, the structure of Rab7 compartments was unaffected upon Synj1 overexpression (Figure 6C). In addition, we monitored the trafficking of Tf receptor in Synj1-overexpressing cells (Figure 7), performing the same assay used for fibroblasts (Figure 4A). After the pulse, Tf trafficking was remarkably delayed in Synj1-overexpressing cells with a consequent great accumulation of Tf inside the Synj1-overexpressing cells versus wild-type cells as revealed at chase time points (Figure 7). Moreover, quantitative analysis clearly showed that the kinetics of Tf trafficking were striking different (Figure 7), thus indicating the perturbation of its recycling.

Altogether, these results indicate that the overexpression of Synj1 impairs the homeostasis of EEs and perturbs the recycling trafficking, thus suggesting that the endosomal alterations observed in DS are related to an increased SYNJ1 dosage.

### 3.5 Alterations of the Lysosomal Compartment in DS Fetal Fibroblasts

As mentioned above, the endolysosomal pathway relies on a highly dynamic network of interacting compartments. Hence, at this point, we asked whether the later station of this pathway could be altered in DS. Therefore, we looked at the distribution and dynamics of Lamp-1 and Cathepsin D, a membrane-associated protein and soluble lysosomal protein, respectively.

**FIGURE 8:**
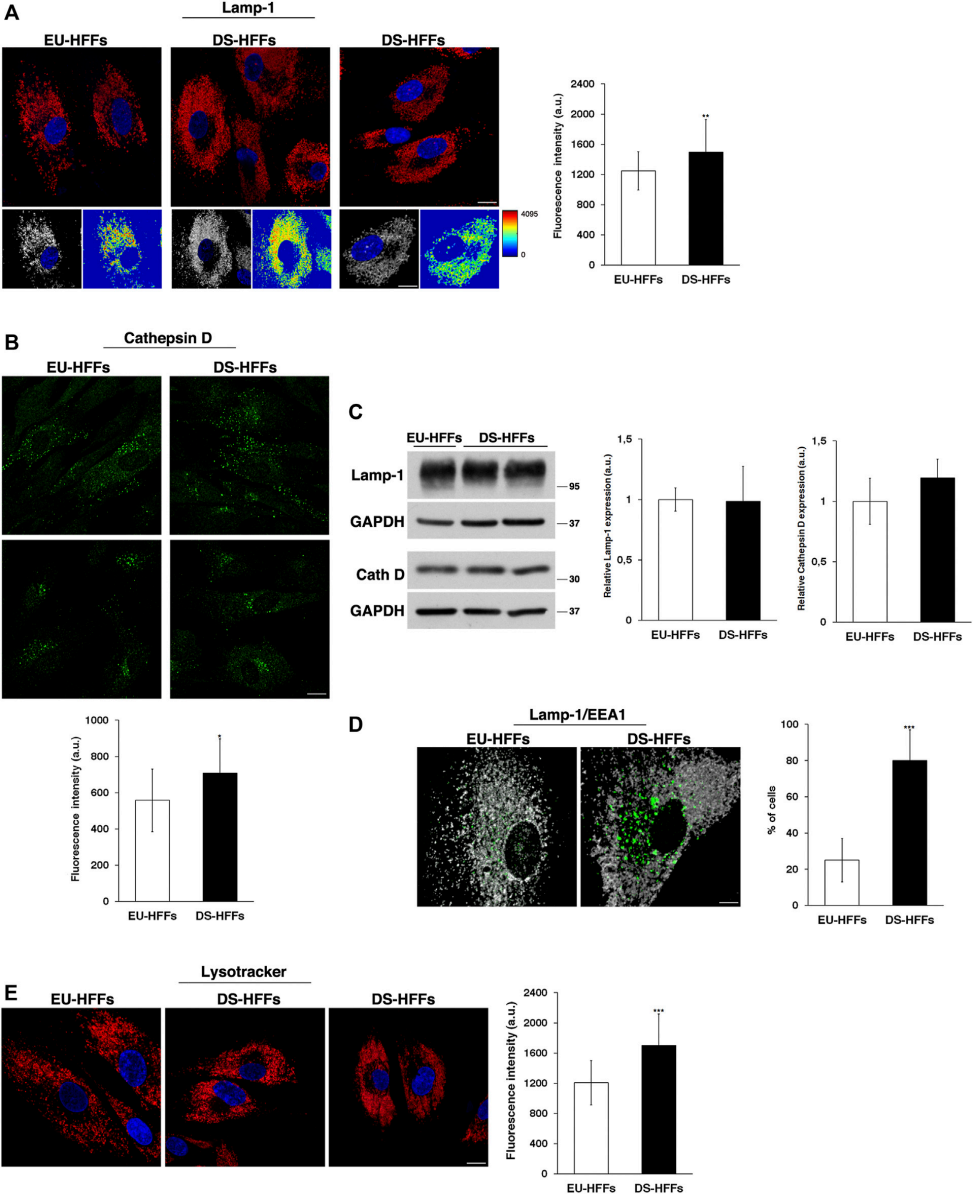
The structure and distribution of lysosomes are perturbed in DS-HFFs. **(A,B)** Immunofluorescence assays using Lamp-1 **(A)** and Cathepsin D **(B)** antibodies in EU-HFFs and DS-HFFs are shown. Serial confocal sections were collected from the top to the bottom of the cells; scale bars, 8 μm. The 3D reconstruction and corresponding intensity map are shown for Lamp-1 staining. Mean fluorescence intensity (arbitrary unit, a.u.) is shown. The bars show the relative mean value ± SD of three independent experiments, *n* ≥ 50 cells. **(C)** Representative immunoblotting of Lamp-1 and Cathepsin D in DS-HFFs and EU-HFFs (longer exposure of the immunoblot is shown in Supplementary Figure S3). GAPDH was used as the loading control. The molecular weight of protein markers is indicated. Densitometric analysis of three different experiments is shown. **(D)** Double immunostaining of Lamp-1 and EEA1 in EU-HFFs and DS-HFFs is shown; scale bar, 8 μm. Representative 3D reconstruction is also shown; scale bar, 4 μm. The graph shows the percentage of the cells displaying lysosome delocalization. **(E)** Lysosomes of DS-HFFs and EU-HFFs were labeled by using LysoTracker dye, which was added to living cells for 1 h at 37°C. Representative images are shown; scale bars, 8 μm. Mean fluorescence intensity (arbitrary unit, a.u) of LysoTracker labeling is higher in DS-HFFs than in EU-HFFs. Error bars, mean ± SD. *p < 0.05, **p < 0.01, ***p < 0.001, Student’s *t*-test.

Lamp-1-positive structures had comparable structure and size in EU-HFFs and DS-HFFs, but they displayed a significantly higher fluorescence intensity in trisomic cells when compared with that in euploid cells (Figure 8A), indicating an alteration of the lysosomal compartment. Cathepsin D immunolabeling showed some heterogeneity both in euploid and trisomic cells - some cells displayed many more positive structures than others (Figure 8B), possibly due to differences in their metabolic state. Nevertheless, Cathepsin D fluorescent dots had a more intense signal in DS-HFFs (Figure 8B), supporting the fact that lysosomes are altered in DS. However, no significant differences in the levels of Lamp-1 and Cathepsin D were detected by Western blot analysis (Figure 8C and Supplementary Figure S3).

In addition, we observed, in trisomic cells, a striking delocalization of lysosomes, which were far from the nucleus (Figures 8A,D,E), leaving an empty perinuclear region. The percentage of cells displaying this altered distribution was significantly higher in DS-HFFs with respect to EU-HFFs (Figure 8D). Interestingly, double immunofluorescence assays using Lamp-1 and EEA1 antibodies showed that the cell area devoid of lysosomes was occupied by large EEA1-positive structures (Figure 8D), suggesting that the altered homeostasis of EEs affects the perinuclear lysosome distribution.

Finally, by labeling lysosomes with LysoTracker, a fluorescent acidotropic dye, we found a significant increase of fluorescent signals in DS-HFFs compared with EU-HFFs (Figure 8E). This might be correlated with changes in the luminal pH, which has been found to depend upon the position of lysosomes (Johnson et al., 2016).

**FIGURE 9:**
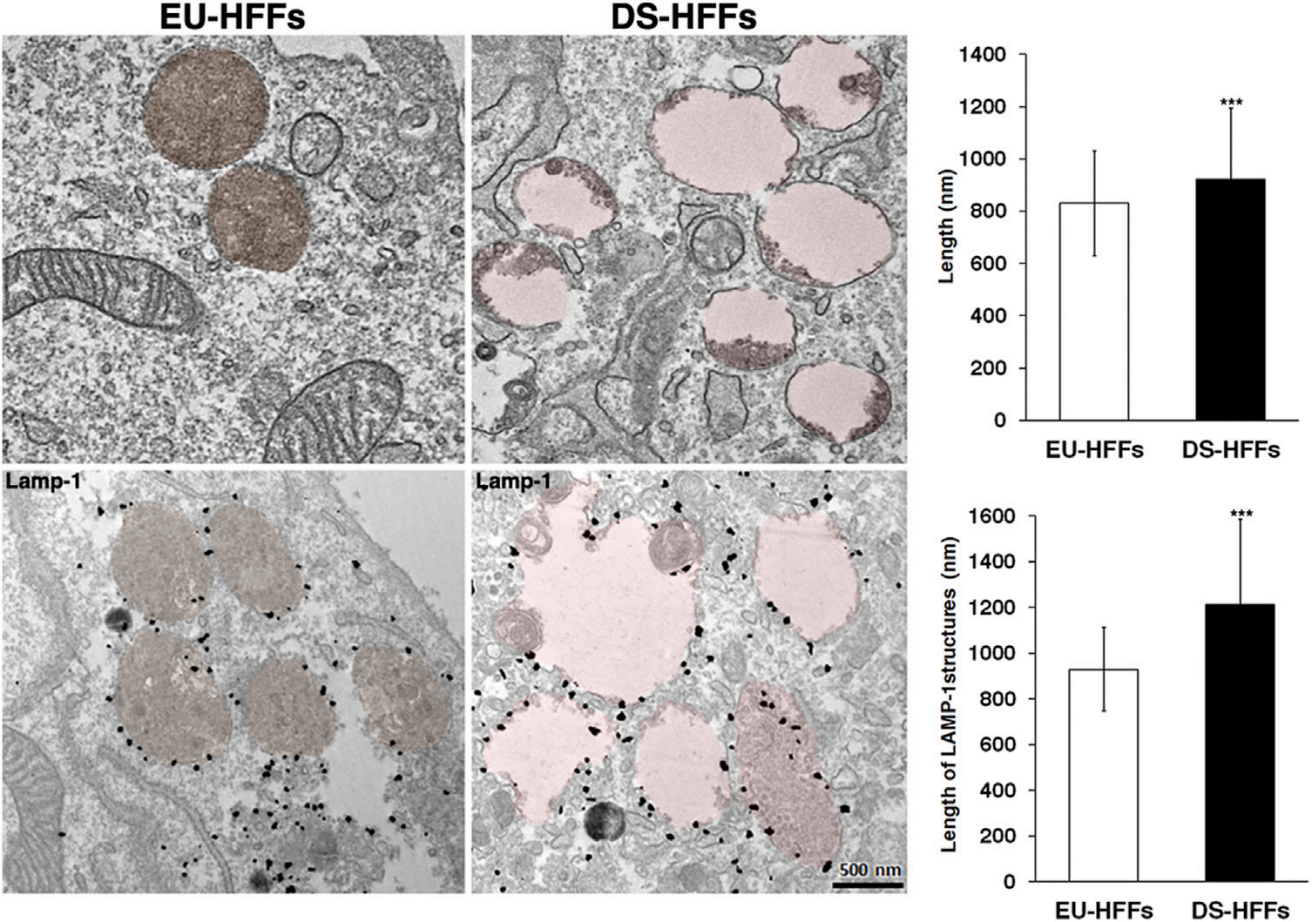
Ultrastructure of endolysosomal compartments is altered in DS fetal fibroblasts. Representative EM and immune-EM of EU-HFFs and DS-HFFs labelled with an antibody against Lamp-1 antibody. Lysosomal-like structures are depicted in brown (control cells) and pink (DS cells) by false colours. The length of lysosome-like structures in EU-HFFs and DS-HFFs is shown. The bars show the relative mean value ± SD of three (EM) ad two (immuno-EM) independent experiments of two euploid and three trisomic cultures, *n* ≥ 20 cells. ***p < 0.001, Student’s *t-test*.

Consistently, ultrastructure analysis showed a striking enlargement of the endolysosomal compartment in trisomic fibroblasts (Figure 9). Interestingly, most of the enlarged structures in DS fibroblasts were Lamp-1 positive, supporting that lysosomes are altered in DS-HFFs (Figure 9).

Taken together, all of these findings point to an alteration of lysosomal compartments in DS.

**FIGURE 10:**
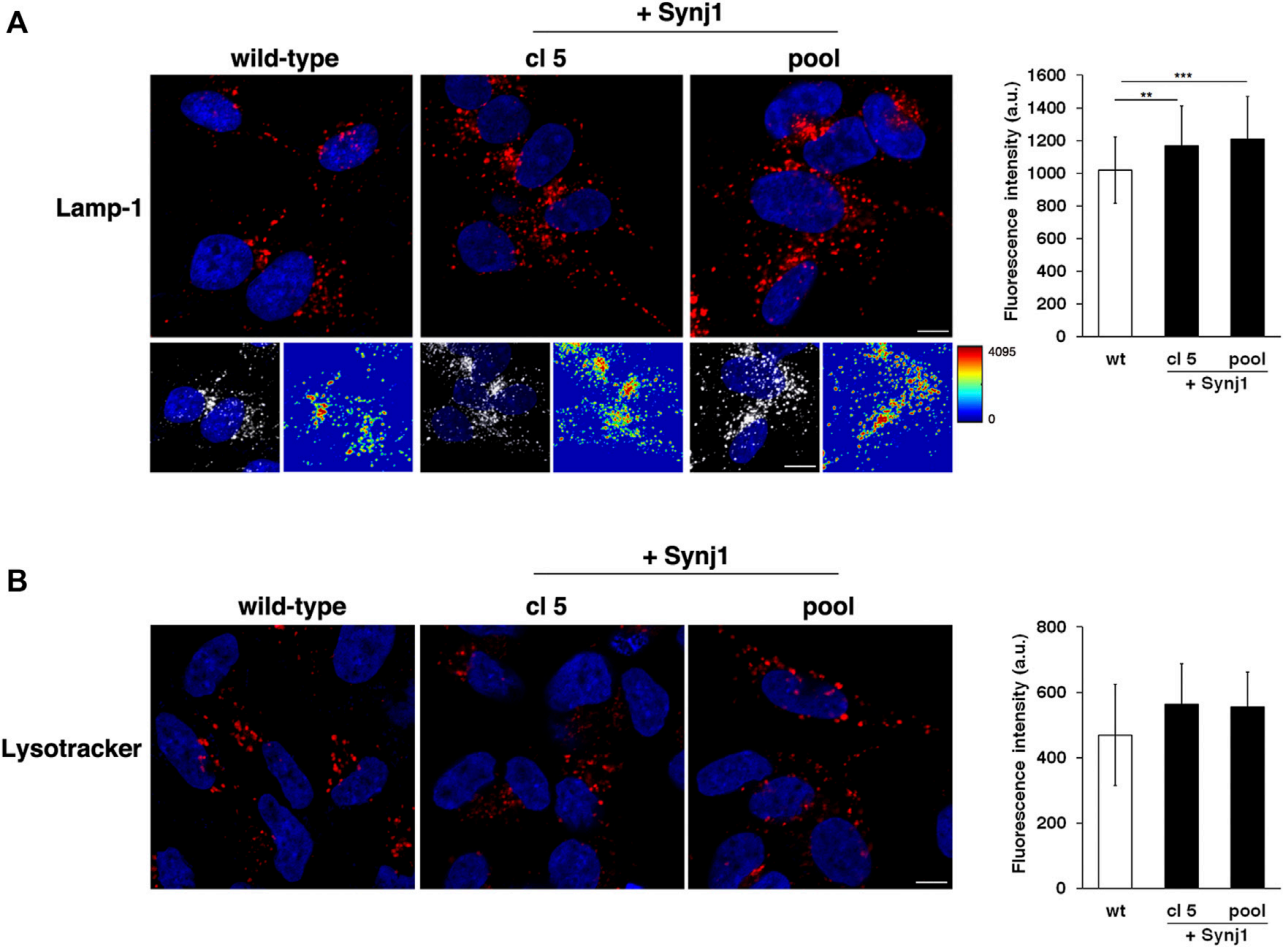
Synj1 overexpression affects the homeostasis of lysosomal compartment in SH-SY5Y cells. **(A)** Immunofluorescence assays using Lamp-1 antibody in wild-type and Synj1-overexpressing SH-SY5Y cells, carried out as in Figure 8A, are shown. Serial confocal sections were collected from the top to the bottom of the cells. The 3D reconstruction and corresponding intensity map are shown. Scale bars, 8 μm. The bars show the relative mean value ± SD of three independent experiments, *n* ≥ 50 cells. **(B)** Lysosomes of wild-type and Synj1-overexpressing SH-SY5Y cells were labelled with LysoTracker dye as in Figure 8E. Serial confocal sections are shown; scale bars, 8 μm. The bars show the relative mean value ± SD of three independent experiments, *n* > 40 cells. **p < 0.01, ***p < 0.001, Student’s *t*-test.

Furthermore, it is interesting that SYNJ1 overexpression affects lysosome structures although at a lesser extent than observed in DS fibroblasts. In Synj1-overexpressing SH-SY5Ycells Lamp-1 positive structures displayed higher fluorescence intensity with respect to wild-type cells (Figure 10A), indicating an alteration of this compartment. No significant difference was detected for Lysotracker staining (Figure 10B).

## 4 Discussion

**FIGURE 11:**
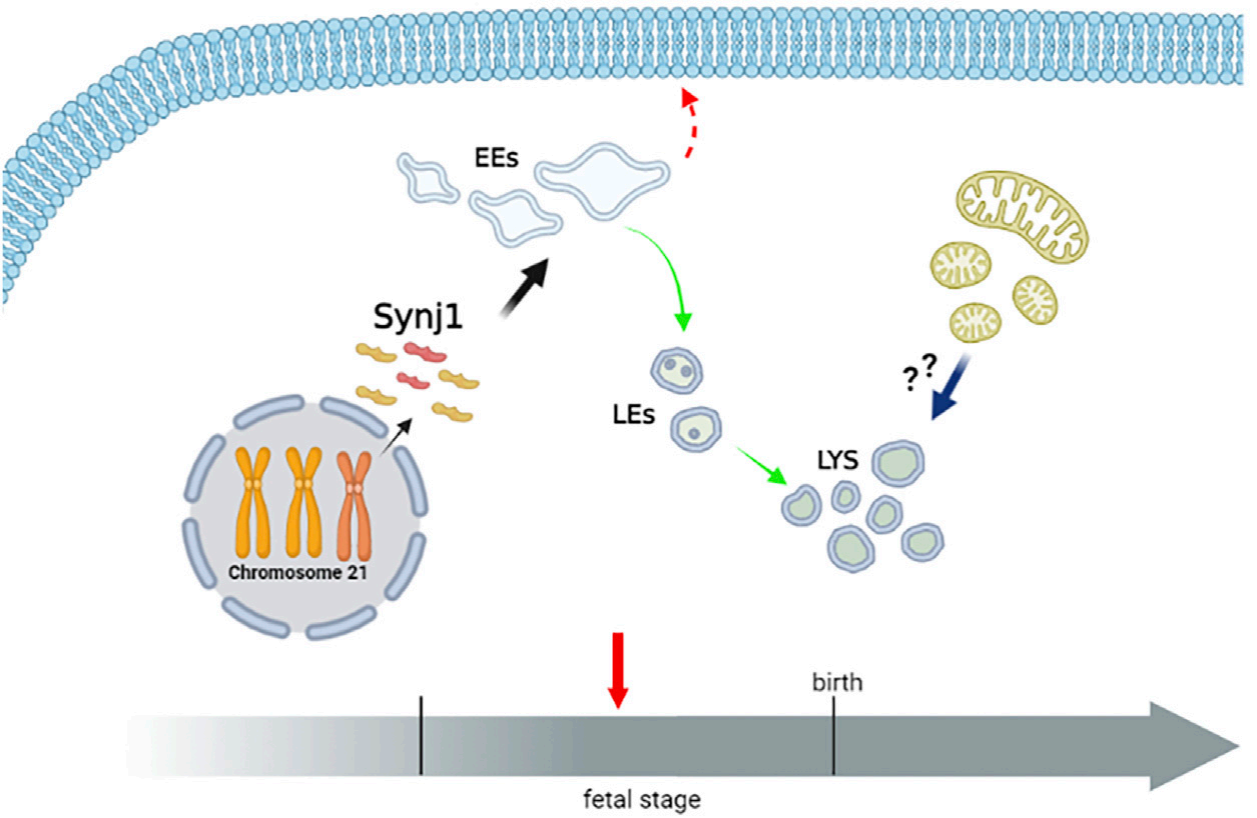
Perturbation of endosomal trafficking is altered early in Down syndrome. The scheme recapitulates the alterations of the endolysosomal pathway observed in DS fetal fibroblasts. The homeostasis of early, but not late, endosomal compartments is altered in DS cells. Moreover, the recycling pathway to the cell’s surface is perturbed, while the trafficking toward the lysosomes is unaffected. Lysosomal compartment is slightly altered in DS cells. Whether lysosomal alterations reflect a compensatory mechanism consequent to the altered dynamics of EEs, an increased autophagy/mitophagy pathway or both remain important intriguing open questions. Hence, a dysfunctional endolysosomal pathway might contribute to the pathogenesis of DS, and Synj1, whose expression is already increased in the fetal stage, may play a potentially critical role (Created with BioRender.com).

The complex multiorgan phenotype of DS reflects the perturbation of multiple pathways at the cellular level. This study indicates that the dysfunctional endolysosomal pathway may concur to the pathogenesis of DS and also points out the potential critical role of Synj1 in DS (Figure 11).

### 4.1 Role of the Endolysosomal Pathway in DS

Here, we show that the structure and dynamics of EEs, but not of late endosomes, are strikingly altered in DS cells. Moreover, the recycling pathway of TfR to the plasma membrane is impaired, thus indicating that endosomal trafficking is perturbed in DS cells.

The accumulation of abnormal early endosomal structures has also been reported in cells from adult individuals with DS (Cataldo et al., 2008; Cossec et al., 2012; Botté et al., 2020), as well as in the most commonly used mouse model of DS, Ts65Dn (Cataldo et al., 2003), thus overall indicating the critical role of the endosomal pathway in DS. Remarkably, our data indicate that perturbation of the endosomal trafficking may represent an early event in the pathogenesis of DS, as we have demonstrated that it occurs in fibroblasts derived from early gestational age fetuses as early as 18 weeks. An important implication is that dysfunction of the endosomal pathway may have multiple effects, likely on different organs, during development.

Endosomal trafficking is crucial for cellular homeostasis. It plays an essential role in controlling the localization and levels of a multitude of proteins and, in turn, ensures the maintenance of homeostasis and functions of endolysosomal compartments. Such trafficking pathways are critical for neuron viability and functions as evidenced by the number of genetic defects in the trafficking machinery that have been associated with neurological disorders (Wang et al., 2014; Schreij et al., 2016; Yarwood et al., 2020). Dysfunction of endosomal trafficking can lead to altered localization or levels of specific signaling receptors, thus disrupting signaling cascades that are required for neuronal survival. Impaired retrograde transport of nerve growth factor (NGF) and consequent failure of its signaling has been shown to cause cholinergic neuron degeneration in Ts65Dn mice (Cooper et al., 2001; Salehi et al., 2006), emphasizing the role of the endosomal pathway for neuronal viability. Moreover, EEs, which are the central sorting station along the endocytic pathway, are crucial for neurons because they ensure the fine balance between recycling and degradation of synaptic proteins and/or other cargoes (such as neurotransmitters, growth factor receptors, and transporters). Inhibition of recycling to the cell surface of glutamate receptors with a corresponding increase of their degradation in Ts65Dn mice has been reported (Wang et al., 2013), providing further evidence for the implication of altered endosomal trafficking in DS neuropathogenesis.

Furthermore, endosomal trafficking supplies the bulk membrane flow that is required for continuous neurogenesis (Zakharenko and Popov, 2000). Therefore, alterations of such pathway may contribute to the decreased dendritic arborization and altered synaptogenesis observed in both prenatal and in postnatal DS brains (Becker et al., 1986; Weitzdoerfer et al., 2001).

All these findings strongly suggest that the dysfunction of this critical hub may be a neuropathological mechanism of disease. The fact that alterations in the endosomal pathway occur early in DS suggests that the dysfunction of this pathway may account for the neurodevelopmental anomalies observed in DS (Antonarakis et al., 2020). Taking into account recent advancements in the experimental procedures to differentiate induced pluripotent stem cells derived from subjects with DS (Hibaoui et al., 2014; Mollo et al., 2021), it will be very interesting to test this hypothesis.

However, dysfunction of the endolysosomal pathway may contribute to other defects in individuals with DS. A recent study showing altered trafficking of immunoglobulins in DS fibroblasts (Cejas et al., 2021) supports a correlation between dysregulation of endosomal trafficking and immune dysregulation and susceptibility to infections observed in DS (Antonarakis et al., 2020; Dieudonné et al., 2020). Moreover, compelling evidence has shown that endosomal transport is critical for heart functions (Curran et al., 2015), suggesting a potential role of this pathway in the cardiac defects of DS.

In addition, we observed alterations in lysosomal compartments in fetal DS fibroblasts, although less dramatic than those revealed for EEs. However, the significant number of enlarged Lamp-1 positive structures observed in DS fibroblasts by IEM analysis further support the alteration of lysosomes in trisomic cells. This could be a consequence of the altered dynamics of EEs. The increased levels of Rab5 and its effector EEA1, both crucial for the dynamics of EEs (Mills et al., 1998; Zeigerer et al., 2012; Murray et al., 2016) and their stalling on EEs may perturb the fine balance of membrane flows among endosomal compartments. Likely, the increased lysosomal biogenesis may be activated as a compensatory mechanism. Further studies will be important to test this hypothesis.

Such lysosomal alterations could also reflect a different degradative behavior of the cells. Consistent with this hypothesis, an increase in the autophagy pathway of DS cells has been reported (Colacurcio et al., 2018). Further support comes from our observations of an increased number of autophagosomes in DS fetal fibroblasts (data not shown). Moreover, the decline in mitochondria quality in DS (Mollo et al., 2020) may induce hyperactivation of the mitophagy pathway. In addition, some findings have provided evidence that the rise of APP-βCTF (the β-cleaved carboxyl terminal fragment), consequent to APP triplication, leads to lysosomal dysfunction (Colacurcio et al., 2018; Jiang et al., 2019).

Furthermore, the delocalization of lysosomes that we observed may also negatively affect lysosome function, since their location correlates well with their degree of acidification (Johnson et al., 2016), which is essential for hydrolase activity. This altered distribution could be the consequence of the collapse of enlarged EEs as supported by the fact that EEs occupy the perinuclear region devoid of lysosomes (Figure 8). Of note, after the initial discovery of membrane contact sites between the ER and mitochondria, it is becoming clear that mitochondria can establish functional contacts with other organelles, including endosomes and lysosomes (Scorrano et al., 2019). Interestingly, it was recently reported that mitochondrial oxidative stress, one of the cellular features of DS cells, increased the number of mitochondria and endosomes engaged in membrane contact sites and altered their dynamics (Hsu et al., 2018). In light of these recent findings, our data and the widely documented mitochondrial dysfunction in DS (Izzo et al., 2018; Valenti et al., 2018; Mollo et al., 2021), the existence of dangerous cross-talk between the endolysosomal system and mitochondria in the pathogenesis of DS is conceivable.

### 4.2 Role of Synj1 in DS

Synj1 is a key player in early endosomal compartments, regulating their homeostasis and functions in different human cell types, including neuronal cells (Fasano et al., 2018). Loss of Synj1 activity leads to the enlargement of EEs and impairment of recycling trafficking (Fasano et al., 2018).

Here, we have shown that the overexpression of Synj1 in SH-SY5Y cells affects the structure and dynamics of EEs, recapitulating the same phenotype seen in DS fetal fibroblasts where Synj1 expression is increased. Our data strengthen the crucial role of Synj1 in modulating endosomal trafficking on the one hand, and they highlight potential Synj1 involvement in the pathogenesis of DS on the other hand. A meta-analysis performed on 45 DS datasets revealed that SYNJ1 is among the top 30 Hsa21 genes consistently upregulated in trisomic cells and tissues (Vilardell et al., 2011), further supporting its role in DS. Remarkably, the finding that the attenuation of Synj1 expression restores the EE morphology indicates that the increased dosage of Synj1 contributes to the EE alterations observed in DS and further corroborates the Synj1 role in DS pathogenesis.

Interestingly, the upregulation of the *Drosophila* homolog of human SYNJ1 alters synaptic function (Chang and Min, 2009). Mice overexpressing Synj1 showed defects in performing the water maze task, exhibiting learning and memory deficits (Voronov et al., 2008; Miranda et al., 2018). Consistently, these defects were rescued by restoring the Synj1 disomy (Voronov et al., 2008). Taken together, these findings provide evidence that Synj1 might contribute to intellectual disability in individuals with DS. Alterations in phosphoinositide homeostasis caused by the increased dosage of Synj1 may contribute to neuronal dysfunction. Consistent with this hypothesis, decreased levels of PI4,5P2, one of the Synj1 substrates, were found in the brains of DS mice (Voronov et al., 2008). Phosphoinositides, which are implicated in many aspects of cellular physiology (Di Paolo and De Camilli, 2006; Balla, 2013), have emerged to play a critical role in the nervous system, and their levels need to be finely regulated (Raghu et al., 2019). Of note, another Synj1 substrate, early endosomal PI3P, is implicated in controlling the activity of neurotransmitter receptors (Menzies et al., 2017; Papadopoulos et al., 2017). Consistently, low levels of this PI have been correlated to defective myelination and reduction of synaptic activity (Wang et al., 2011; Logan et al., 2017). Thus, as a consequence of excessive Synj1 activity, it is likely that the altered metabolism of Synj1-phosphoinositides may have a negative impact on neuronal function and therefore account for some neuronal anomalies in DS.

Remarkably, PI3P contributes to controlling the dynamics and functions of EEs, thus suggesting that PI3P imbalance, caused by the excessive Synj1 activity, could be responsible for the endosomal trafficking defects observed in DS. The recent finding showing the decreased levels of PI3P in adult fibroblasts supports this hypothesis (Botté et al., 2020). Hence, a dangerous liaison among Synj1, endosomal trafficking and DS is conceivable.

It is intriguing that suppression and overexpression of SYNJ1 lead to apparently similar effects on EEs. However, although we detected enlargement/swelling of these compartments under both conditions, the nature and molecular content of these enlarged/swelled endosomes and therefore their nature might be different. Moreover, our findings raised a subtle but important difference in terms of trafficking: whereas the recycling of transferrin is completely impaired upon Synj1 downregulation and transferrin accumulates in the pericentriolar region (Fasano et al., 2018), it is striking perturbed, rather strongly delayed, upon Synj1 overexpression. In addition, transferrin-positive compartments appear throughout the cell in DS fibroblasts suggesting that the fast recycling pathway (Zerial and McBride, 2001), which appears to be the main route in these cells in light of the Tf kinetics detected in euploid cells, is altered.

Overall, all of these data clearly indicate that EE dynamics are altered when Synj1 levels, and plausibly its activity, are unbalanced but probably in different manner.

In this context, the PI3P levels are critical and its dyshomeostasis can have several effects. On the one side, the increased PI3P levels may promote excessive recruitment on EEs of proteins bearing PI3P-binding sites, such as EEA1, inducing their stalling on compartments, which in turn may affect the dynamics and functions of the compartment itself. On the another side, recent data have shown that PI3P is crucial in modulating Rab5 activity (Cezanne et al., 2020). In particular, *in vitro* studies using supported lipid bilayers unraveled that PI3P is required for further recruitment of Rab5 and for formation of Rab5-nanodomains that have been shown to be functional for initiating vesicle docking and fusion (Murray et al., 2016; Franke et al., 2019; Cezanne et al., 2020). Thus, increased amounts of PI3P can be expected to promote vesicle tethering and fusion by expanding the compartment due to an imbalance between membrane entry and exit; whereas an arrest in a tethered state rather than fusion may be induced by the low PI3P levels. Further studies are necessary to elucidate these aspects. Moreover, the scenario is more complicated by the fact that endosome membranes have a self-organization based on the dynamic and cooperative work of their molecular components, such as Rab5 and EEA1, as pointed out by the Zerial’s group (Zeigerer et al., 2012; Murray et al., 2016; Franke et al., 2019; Lauer et al., 2019; Cezanne et al., 2020). Hence, altering the biomechanical properties of endosomal membranes can impact, in a non-linear way, the different pathways in which these organelles are involved. Loss of Rab5 below a critical threshold causes reduction of the entire endolysosmal system as well as an impairment of endocytosis (Zeigerer et al., 2012), while the GTPase-deficient Rab5 mutant leads to enlargement of early and late compartments (Wegener et al., 2010). Along these lines, Steinfeld and others, by analyzing the effects of yeast phosphatidylinositol 3-Kinase (PI3K) Vps34 hyperactivation, have provided evidence that the high PI3P levels did not affect ESCART pathway but slowed the autophagy, despite both depending on PI3P (Steinfeld et al., 2021). On the other hands, it has been shown that the loss of VPS34 causes an accumulation of enlarged late endosomes and negatively regulates the late endocytic trafficking in mouse embryonic fibroblasts (Jaber et al., 2012; Jaber et al., 2016). Conditional KO mice with sensory neuron-specific deletion of VPS34 displayed two populations of sensory neurons with a different phenotype in terms of degree and nature of endosomal alterations (e.g., large vesicles of transparent appearance vs. filled enlarged lysosomes) (Zhou et al., 2010), suggesting that alterations of molecular machinery regulating PIs may be cell-context dependent.

Overall, it is clear that the imbalance of PI3P is critical for endosomal pathway and, in turn, for cellular homeostasis, and like for other PIs its concentration has to be precisely regulated in space and time.

Together with previous findings, our study highlights that adequate Synj1 levels are critical for cellular physiology, implying that regulation of its expression and/or activity is crucial. This is true, in particular, for neuronal cells. Interestingly, three known regulators of Synj1 activity, Dyrk1A, RCAN1 and intersectin (Cousin and Robinson, 2001; Koh et al., 2004; Adayev et al., 2006), map to chromosome 21, making the role of Synj1 in the pathogenesis of DS more intriguing. Of note, like Synj1, Dyrk1A, a serine-threonine kinase that phosphorylates Synj1 and RCAN1, a regulator of calcineurin implicated in Synj1 dephosphorylation, are among the Hsa21 genes consistently up-regulated in trisomic cells and tissues (Vilardell et al., 2011). In conclusion, the previous findings and ours reveal the critical importance of Synj1 in the pathogenesis of DS. Further studies will be important to gain more insight into the contribution of Synj1 to DS.

## Data Availability

The original contributions presented in the study are included in the article/Supplementary Material, further inquiries can be directed to the corresponding authors.
