# Supplementary material for: Down Syndrome Fetal Fibroblasts Display Alterations of Endosomal Trafficking Possibly due to SYNJ1 Overexpression
Source: Front Genet. 2022 May 13;13:867989. doi: 10.3389/fgene.2022.867989 (PMC9136301; doi:10.3389/fgene.2022.867989)
Supplement: Supplementary file 1 [file DataSheet1.pdf]

Supplementary Material

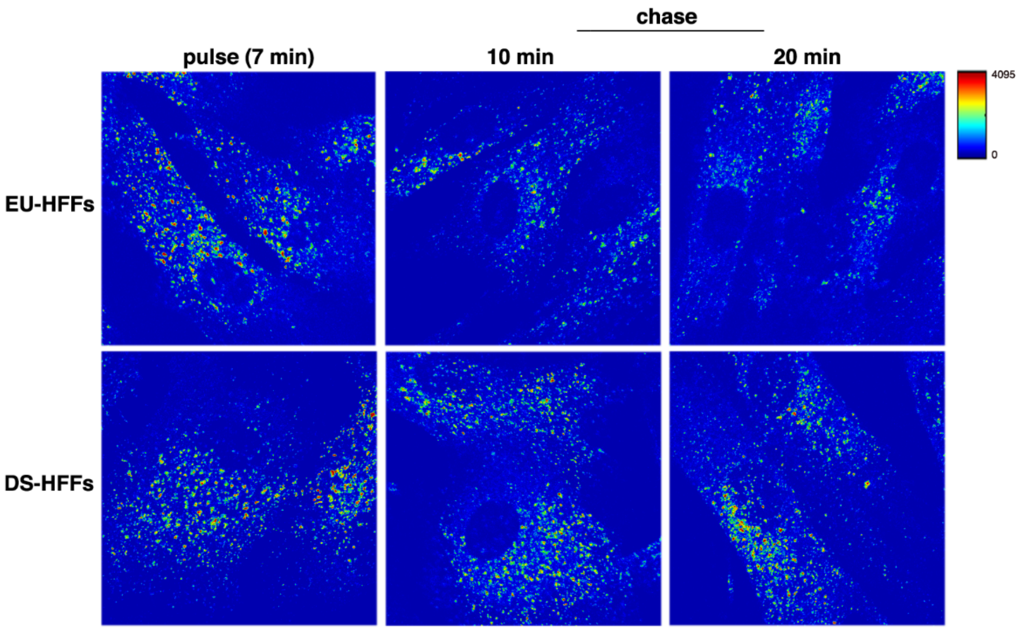

Supplementary Figure 1. TF recycling in DS fetal fibroblasts. Note that the fluorescence signal decreases in EU-HFFs, while remains higher in DS-HFFs indicating TF accumulation.

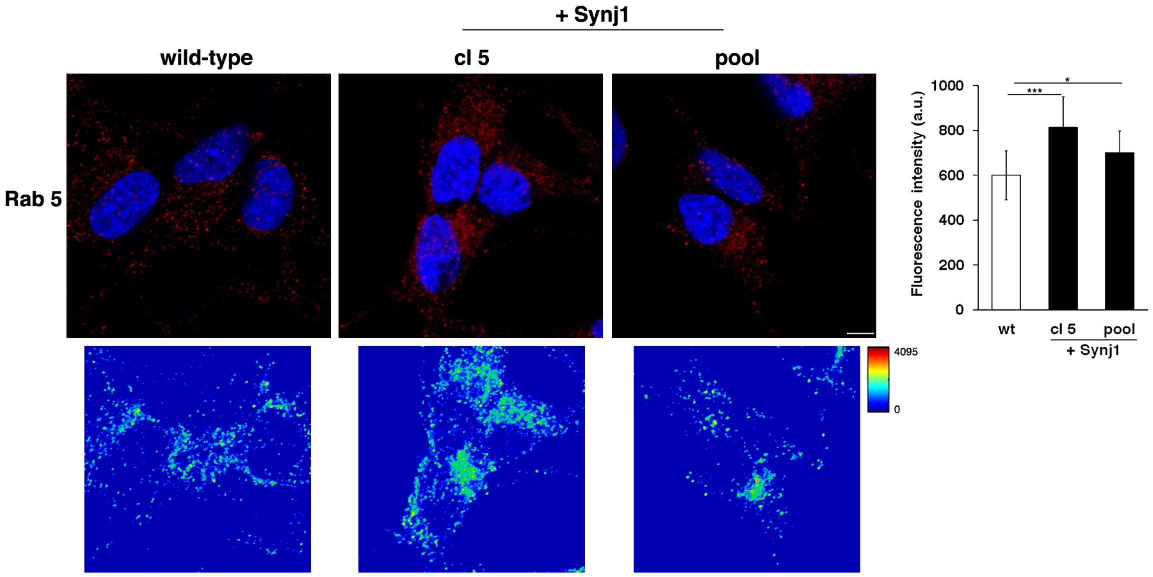

Supplementary Figure 2. Synj1 overexpression affects the homeostasis of early endosomes in SH-SY5Y cells. SH-SY5Y cells stably transfected with cDNA encoding for wild-type Synj1 were stained with Rab5 antibody detected with Alexa-546-conjugated secondary antibodies. Serial confocal sections were collected from the top to the bottom of the cells. The 3D reconstructions and corresponding intensity maps are shown. Scale bars, 5 µm. Mean fluorescence intensity (arbitrary unit, a.u.) of Rab5-positive structures is shown. The bars show the relative mean value ± SD of three independent experiments. *$p < 0.05$, ***$p < 0.001$, Student's *t* -test.

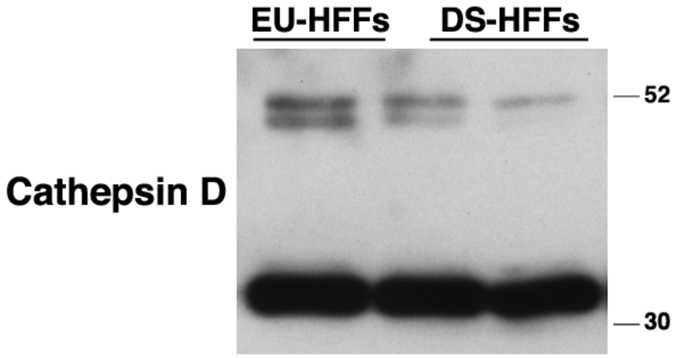

Supplementary Figure 3. Levels of cathepsin D are comparable in EU-HFFs and DS-HFFs. The longer exposure of immunoblot in Figure 8C is shown. Mature (33 kDa) and immature (52 kDa) forms of cathepsin D are visible.
